# Editorial: The impact of adipose tissue dysfunction on cardiovascular and renal disease, Volume II

**DOI:** 10.3389/fendo.2023.1223983

**Published:** 2023-06-14

**Authors:** Xiaodong Sun, Chengchao Ruan, Alexandre A. da Silva

**Affiliations:** ^1^ Department of Endocrinology and Metabolism, Affiliated Hospital of Weifang Medical University, Weifang, China; ^2^ Clinical Research Center, Affiliated Hospital of Weifang Medical University, Weifang, China; ^3^ Shanghai Key Laboratory of Bioactive Small Molecules, Department of Physiology and Pathophysiology, School of Basic Medical Sciences, Fudan University, Shanghai, China; ^4^ Department of Physiology and Biophysics, Mississippi Center for Obesity Research, and Cardiorenal and Metabolic Diseases Research Center, University of Mississippi Medical Center, Jackson, MS, United States

**Keywords:** cardiovascular disease, chronic kidney disease, diabetic kidney disease, adipose, perivascular adipose tissue, epicardial adipose tissue, adipocytes, obesity

Obesity is a global health problem that affects millions of people and increases the risk of various chronic diseases, such as cardiovascular disease (CVD) and chronic kidney disease (CKD). Adipose tissue is a heterogeneous tissue that regulates metabolism, inflammation and immunity. It also mediates the harmful effects of obesity on health. The location and characteristics of adipose tissue determine its positive or negative impact on different organs and systems (1). Excess adipose tissue mass has been traditionally associated with metabolic dysfunction, but recent evidence suggests that adipose tissue quality and distribution are more critical for metabolic health than quantity (2). In addition to the well-known visceral and subcutaneous fat depots, other adipose tissues around blood vessels, such as perivascular, perirenal and epicardial fat, have emerged as novel contributors to CVD and CKD pathogenesis (3–5). These adipose tissues have unique features and dysfunctions in obesity and metabolic syndrome that may cause vascular and renal damage. However, the molecular mechanisms that mediate the communication between these adipose tissues and the cardiovascular and renal systems are still unclear and need further research. This Research Topic showcases a collection of 7 original research and 4 review articles that span from basic to clinical research, providing new insights into the pathophysiology of adipose dysfunction in CVD and CKD.

Obesity predisposes to various comorbidities. However, the risk of developing these comorbidities differs among obese individuals, depending on fat distribution in different body regions. Liu et al. examined the role of central fat distribution and comorbidity in 4899 obese participants from the NHANES database. They found that more than half had at least one comorbidity, and that central fat distribution varied by sex and age. They also showed that higher android fat ratio, visceral fat ratio and visceral to subcutaneous fat ratio were associated with increased risk of comorbidity in both men and women, while higher gynoid fat ratio and subcutaneous fat ratio were associated with decreased risk of comorbidity. These results suggest that central fat distribution is strongly related to comorbidity in obese individuals.

Perivascular adipose tissue (PVAT) is a fat tissue that wraps around blood vessels and can produce various factors influencing vascular tone, inflammation, and remodeling. One of the mechanisms that may control PVAT function is mechanotransduction, which is how cells sense and respond to mechanical forces. PIEZO1 is a mechanosensitive ion channel protein found in various cell types, including adipocytes. Rendon et al. found that pressure or stretch can activate PIEZO1 in PVAT preadipocytes, affecting multiple cellular processes, such as proliferation, differentiation, migration, and metabolism. These data suggest that PIEZO1 activation in PVAT reduces adipogenesis and lipogenesis and may be an adaptive response to hypertension.

Another fat depot that can modulate CVD is epicardial adipose tissue (EAT), which surrounds the heart and can have both beneficial and detrimental effects on cardiac function and metabolism. EAT can exert cardioprotective and metabolic effects, but it can also induce inflammation and metabolic dysfunction that exacerbate CVD. Li et al. provided a comprehensive overview of the role of EAT in CVD and discussed the methods for EAT quantification and the potential strategies for EAT manipulation. A similar review by Willar et al. summarized how aging and obesity increase EAT size and how EAT is linked to atrial fibrillation and its complications. They proposed that EAT may interact with the cardiac autonomic nervous system and the atrial cardiomyocytes to modulate atrial electrophysiology and arrhythmogenesis. In an original study, Gruzdeva et al. investigated the association of adipocytokines in different fat depots with cardiovascular risk factors in patients with coronary artery disease or valve disease. They reported that low levels of ADIPOQ expression and high levels of interleukin-6 in EAT may increase the risk of atherosclerosis and CAD progression, especially in combination with other risk factors. More research is needed to elucidate the mechanisms and implications of EAT in CVD and to develop effective interventions for EAT modulation.

CKD is another chronic disease that is associated with obesity-related adverse events. Besides the adipose-CVD axis, Arabi et al. provided a comprehensive update on the mechanisms and clinical implications of the adipose-renal axis in CKD. They described how obesity predisposes to CKD and how CKD alters adipose tissue function and exacerbates insulin resistance, creating a feedback loop between the kidney and the fat tissue. This highlights the role of cellular senescence in both adipose tissue and CKD.

Upper-body subcutaneous fat is also a unique fat depot that presents an extra risk for metabolic disorders, estimated by neck circumference and neck-to-height ratio (NHR). He et al. reported that patients with diabetic kidney disease (DKD) had higher neck circumference and neck-to-height ratio (NHR) than those without DKD. They also showed that higher NHR was related to lower estimated glomerular filtration rate (eGFR) and higher albumin-to-creatinine ratio. They concluded that NHR was a risk factor for DKD in this population and suggested that measuring NHR could help identify patients at risk of DKD.

In addition to NHR, other indices that reflect obesity, such as body mass index (BMI) and body roundness index (BRI), also have implications for CKD outcomes, especially in kidney transplant recipients. A cohort study conducted by Bellini et al. studied 396 kidney transplant recipients with different BMI classes and followed them for about 6 years. They found that the recipient’s BMI did not affect the patient’s survival, but it did affect graft survival and function. They concluded that obesity was a risk factor for graft failure and suggested that BMI should be considered when selecting kidney transplant candidates. Similarly, BRI, which is a measure of body roundness based on height and waist and hip circumferences, also showed a negative impact on kidney function in a large Chinese population. Zhang et al. conducted a cross-sectional study of 36,784 Chinese adults aged over 40 years and measured their BRI and eGFR. They reported that higher BRI was associated with low eGFR and this association was stronger in subgroups of older people, women, smokers, and those with diabetes or hypertension. They concluded that BRI was a positive risk factor for kidney disease in the Chinese population and recommended BRI as a screening tool to identify kidney disease complications.

Another specific type of kidney disease that is influenced by obesity is IgA nephropathy, which is characterized by the accumulation of IgA antibodies in the kidney. Wang et al. examined 1054 IgA nephropathy patients and compared their outcomes according to their body weight status. They found that obese IgA nephropathy patients had impaired kidney function, more metabolic disturbances and unhealthy behaviors than non-obese IgA nephropathy patients. They concluded that obesity is a risk factor for IgA nephropathy patients when coexisting with hypertension.

Obesity is also associated with increased inflammation, which can worsen kidney damage and accelerate CKD progression. One of the inflammatory mediators that has been implicated in CKD is chemerin, a chemokine that initiates the early immune response. A meta-analysis by Behnoush et al. compared chemerin levels between CKD patients and healthy controls using 8 high-quality studies with 875 participants. They reported that chemerin levels were significantly higher in CKD patients, especially those on hemodialysis, suggesting more inflammation. They inferred that chemerin could be a potential biomarker for CKD and recommended further research to investigate its clinical and pathophysiological role in CKD.

Overall, the articles presented in this Research Topic highlight the crucial role of various adipose tissue dysfunction in CVD and CKD development and progression, and raise a timely question of whether manipulating specific adipose tissue depots may offer a novel target for more effective strategies to prevent and/or treat CVD and CKD.

## Author contributions

All authors listed have made a substantial, direct, and intellectual contribution to the work and approved it for publication.
